# Supplementary material for: Revisiting the cancer microbiome using PRISM
Source: bioRxiv. 2025 Jan 24:2025.01.21.634087. Preprint. [Version 1] doi: 10.1101/2025.01.21.634087 (PMC11785023; doi:10.1101/2025.01.21.634087)

bioRxiv preprint doi: https://doi.org/10.1101/2025.01.21.634087; this version posted January 24, 2025. The copyright holder for this preprint (which was not certified by peer review) is the author/funder, who has granted bioRxiv a license to display the preprint in perpetuity. It is made available under aCC-BY 4.0 International license.

Figure S1. PRISM contamination model features. Boxplots comparing the values of PRISM model features between known contaminant (C) taxa and truly-present (TP) taxa in the CLID datasets. See Methods for specific feature details and formulas. In all panels, boxplots show median (line), 25th and 75th percentiles (box) and 1.5xIQR (whiskers), and points represent outliers. Wilcoxon testing; ****, $p<0.0001$. **(a)** Features related to microbial and gene and product (as reported by BLAST) statistics calculated from BLAST output; fprod, proportion of total products; fuprod, proportion of unique products; fugene, proportion of unique genes; prod_div, products Shannon diversity; gene_div, genes Shannon diversity. **(b)** Statistics per taxon related to the proportion of reads that are uniquely vs. non-uniquely mappable. mb_strand, strandedness (right vs. left) of mapped reads; mb_uniq, number of unique BLAST accession IDs mapped. **(c)** Statistics per taxon calculated from the Kraken2 report. n2, total reads assigned to the clade rooted at the

bioRxiv preprint doi: https://doi.org/10.1101/2025.01.21.634087; this version posted January 24, 2025. The copyright holder for this preprint (which was not certified by peer review) is the author/funder, who has granted bioRxiv a license to display the preprint in perpetuity. It is made available under aCC-BY 4.0 International license.

taxon; n3, number of reads specifically assigned to the taxon; uniq, estimated number of unique k-mers; fmicro, taxon read counts relative to the total reads counts assigned by Kraken to bacteria, fungi, and viruses. **(d)** Proportion of k-mers per taxon that Kraken2 assigned to each phylogenetic class. **(e)** For each taxon, the number of reads (_n), unique k-mers (_u), or species (_n) that Kraken2 assigned to the same phylogenetic lineage for each level of the phylogenetic tree. (**f**) Boxplots show one-tailed Wilcoxon p-values testing for a difference in Kraken2-related classification feature values for the same taxa when they were contaminants vs. truly-present. P-values were adjusted for false-discovery. The red line marks p=0.05. (**g**) We used multiple linear regression models to test for the significance of the Kraken-related classification features while controlling for taxon read abundance and the number of related taxa (belonging to the same class) present in the reference database. Bars indicate the coefficient p-value for the tested feature. The model tested feature ~ taxon type (contaminant vs. truly present) + read count + number of same class taxa in the reference database. The red line indicates p=0.05.

Figure S2. Validating the PRISM score. (a) Boxplots of PRISM scores for the RNA-seq data from cell lines in the absence of known infection, a negative control validation dataset to test for model prediction bias toward over-represented true-positive taxa in its training data. Data are stratified into deciles related to the number of instances in the training data for each true-positive taxon. The red line marks 0.2, the true-positive threshold used in this paper. Boxplots show median (line), 25th and 75th percentiles (box) and 1.5xIQR (whiskers), and points represent outliers. (**b**) Repeating the analysis from (a) but after subsampling the training data to include 15 instances of each contaminant or each truly-present taxon. The new model is then used to predict PRISM scores for the negative control dataset. Taxa are stratified into the same deciles as in (a). Boxplots are as in (a). (**c**) Confusion matrix of PRISM predictions for the data from Fig. 2p. Data are for five species that were common known contaminants (C) in the negative control datasets vs. when they were validated as truly-present (TP) in human infections. (**d**) Swarm plots of PRISM scores for the data from Fig. 2p, stratified by species; p-values indicated Wilcoxon testing. (**e**) Swarm plots of PRISM scores for the data from Fig. 2p, stratified by sequencing technology; p-values indicated Wilcoxon testing. (**f**) Logistic regression modeling of the data from Fig. 2p assessing the significance of the PRISM score vs. read count in distinguishing contaminant vs. truly-present species. (**g**) Confusion matrix of PRISM predictions for the data from Fig. 2o. Data are for 24 species that were uncommon contaminants (C) in the negative control datasets vs. when they were validated as truly-present (TP) in human infections. (**h**) Swarm plots of PRISM scores for the data from Fig. 2o, stratified by sequencing type; p-values indicated Wilcoxon testing. (**i**) Swarm plot of the number of taxa detected in CDC-HAI WGS of human pathogens for taxa that were (yes, n=20) or were not (no, n=28) in our reference database. (**j**) Swarm plots of percent identical base match (pident) from BLAST for taxa that were or were not in the reference database.

Figure S3. Profiling the microbiome in TCGA. (a) Scatter plot of PRISM scores (0=contaminant, 1=truly-present) for all taxa identified in TCGA. Each point is a taxon, which are ordered by their

bioRxiv preprint doi: https://doi.org/10.1101/2025.01.21.634087; this version posted January 24, 2025. The copyright holder for this preprint (which was not certified by peer review) is the author/funder, who has granted bioRxiv a license to display the preprint in perpetuity. It is made available under aCC-BY 4.0 International license.

PRISM score. The red line is the cutoff (PRISM score > 0.2) used to discard likely contaminants. **(b)** Boxplots of total detected vs. truly-present microbial counts/sample (left) and taxa/sample in 8 cancer types in TCGA. Boxplots show median (line), 25th and 75th percentiles (box) and 1.5xIQR (whiskers), and points represent outliers. **(c)** CPTAC samples ordered by number of microbial taxa (top) and number of microbial (reads) detected. The X-axis is scaled to reflect proportion of samples in each cancer type. Sample curves are colored by cancer type. **(d)** Dot-plot of major genera detected in each cancer type. Dots are colored by the proportion of samples in which the genus was detected. **(e)** Boxplots and swarm plots of the number of taxa/sample detected in CPTAC and TCGA. (**f**) Boxplots and swarm plots of the proportion of samples that each taxon was detected in for taxa detected in CPTAC and TCGA. (**g**) Boxplots showing the number of species per sample detected in CLID in poly-A selected (PAS) vs. ribosomal-depletion (RD) RNA-seq. (**h**) Boxplots comparing the proportion of samples in which each taxon in CLID was detected in PAS vs. RD samples. (**i**) Boxplots comparing the relative abundance of contaminants in CLID detected in PAS vs. RD samples. Contaminants are presumed to be present at a similar level of total abundance in these samples. (**j**) Boxplots comparing the diversity of taxon read counts in CLID in PAS vs. RD samples. (**k**) Principal component plot of TCGA colon and head and neck cancers computed based on taxa relative abundances. Each point represents a sample. Points are colored by their sequencing center and shaped by cancer-type.

Figure S4. Detecting microbes in pancreatic cancer using a stricter PRISM score. (a) Boxplots of the days to tumor recurrence (left) and pack years smoked (right) of pancreatic cancer patients whose tumors have (+MB) or do not have (-MB) detectable microbes. Data were assessed with *E. coli* reads removed. Boxplots show median (line), 25th and 75th percentiles (box) and 1.5xIQR (whiskers). Points represent outliers. P-values are from Wilcoxon testing. (**b**) Bar plot indicating the total read counts of species identified in 159 cases of pancreatic cancer in CPTAC using a more stringent PRISM score (CS) of >0.4. Bars are colored by the number of cases in which the species was detected. **(c)** Bar plot of the most common microbial products detected in CPTAC pancreatic cancer. **(d)** Volcano plot of genes with glycoprotein modification. The x-axis is the $\log_2$-fold change of average gene modification level in tumors with detectable microbiome (+MB; n=91) vs. those without (-MB; n=68). The Y-axis indicates the Wilcoxon p-value. **(e)** Bar plot indicating gene ontology (GO) pathways of the genes with significantly different glycoprotein modifications (p<1e-10) in tumors with vs. without detectable microbiome. (**f**) Boxplots of the days to tumor recurrence (left) and pack years smoked (right) of pancreatic cancer patients whose tumors have (+MB) vs. do not have (-MB) detectable microbiome. Boxplots show median (line), 25th and 75th percentiles (box) and 1.5xIQR (whiskers). Points represent outliers. P-values are from Wilcoxon testing.

bioRxiv preprint doi: https://doi.org/10.1101/2025.01.21.634087; this version posted January 24, 2025. The copyright holder for this preprint (which was not certified by peer review) is the author/funder, who has granted bioRxiv a license to display the preprint in perpetuity. It is made available under aCC-BY 4.0 International license.

Figure S1

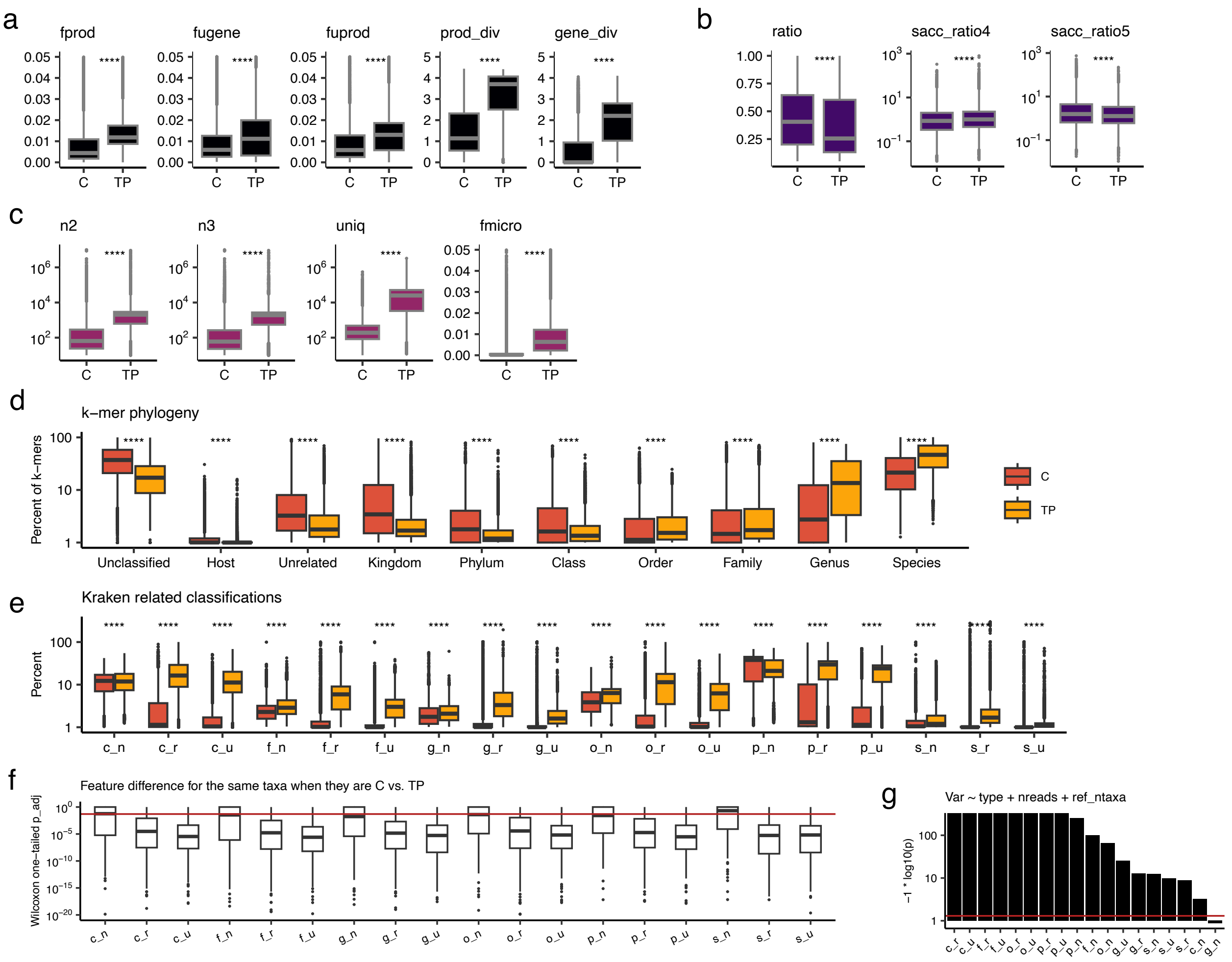

bioRxiv preprint doi: https://doi.org/10.1101/2025.01.21.634087; this version posted January 24, 2025. The copyright holder for this preprint (which was not certified by peer review) is the author/funder, who has granted bioRxiv a license to display the preprint in perpetuity. It is made available under aCC-BY 4.0 International license.

Figure S2

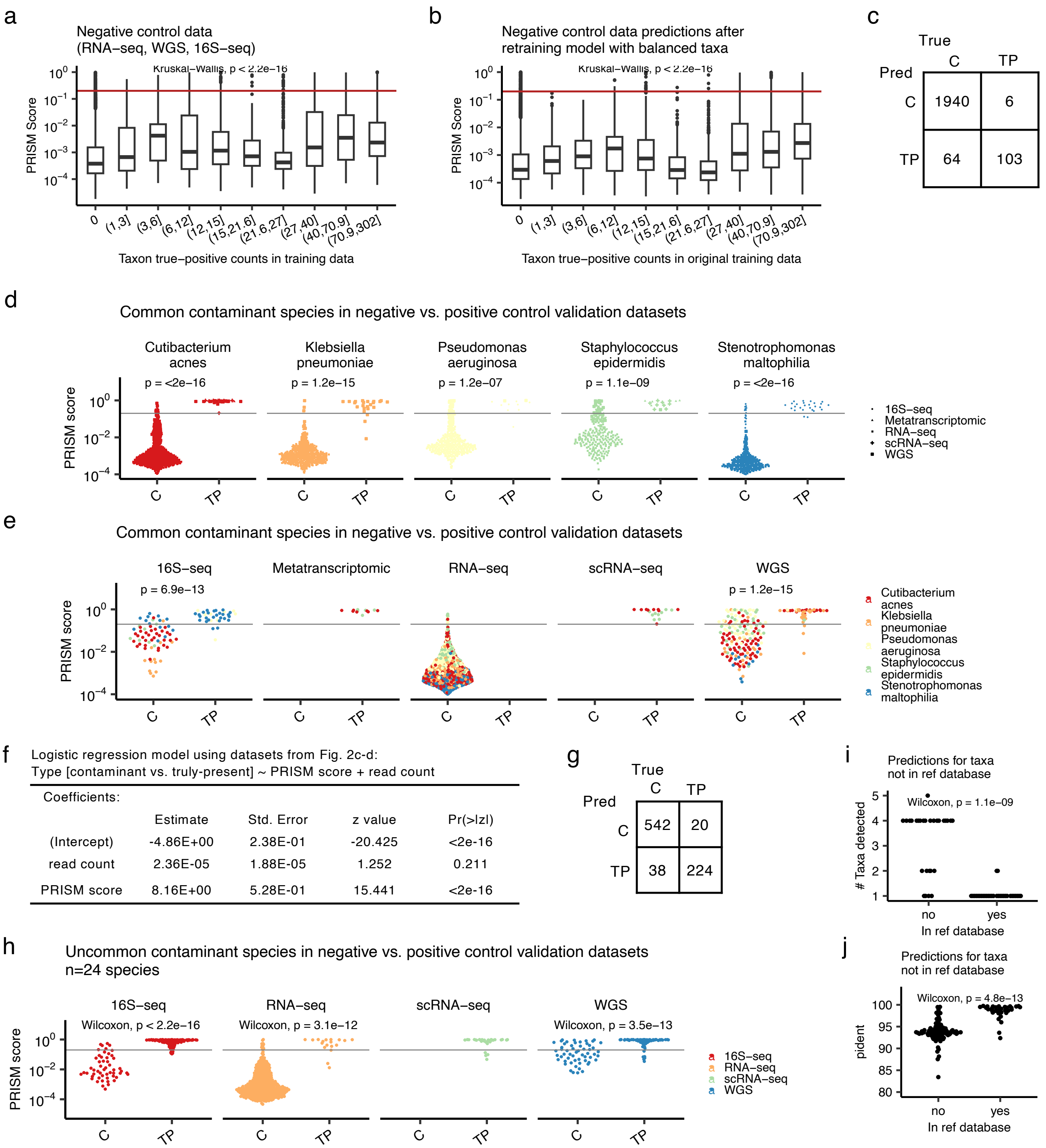

bioRxiv preprint doi: https://doi.org/10.1101/2025.01.21.634087; this version posted January 24, 2025. The copyright holder for this preprint (which was not certified by peer review) is the author/funder, who has granted bioRxiv a license to display the preprint in perpetuity. It is made available under aCC-BY 4.0 International license.

Figure S3

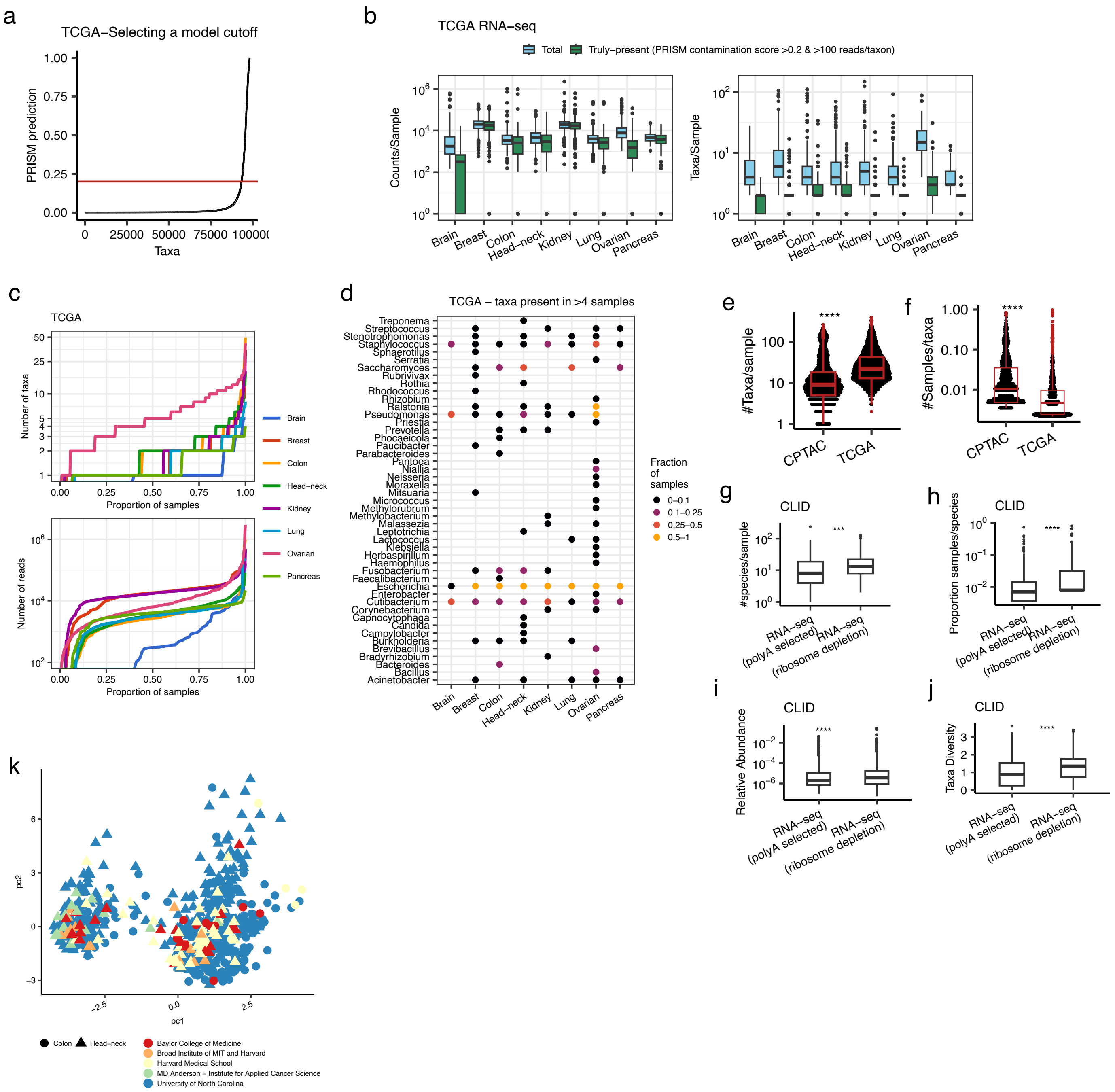

bioRxiv preprint doi: https://doi.org/10.1101/2025.01.21.634087; this version posted January 24, 2025. The copyright holder for this preprint (which was not certified by peer review) is the author/funder, who has granted bioRxiv a license to display the preprint in perpetuity. It is made available under aCC-BY 4.0 International license.

Figure S4

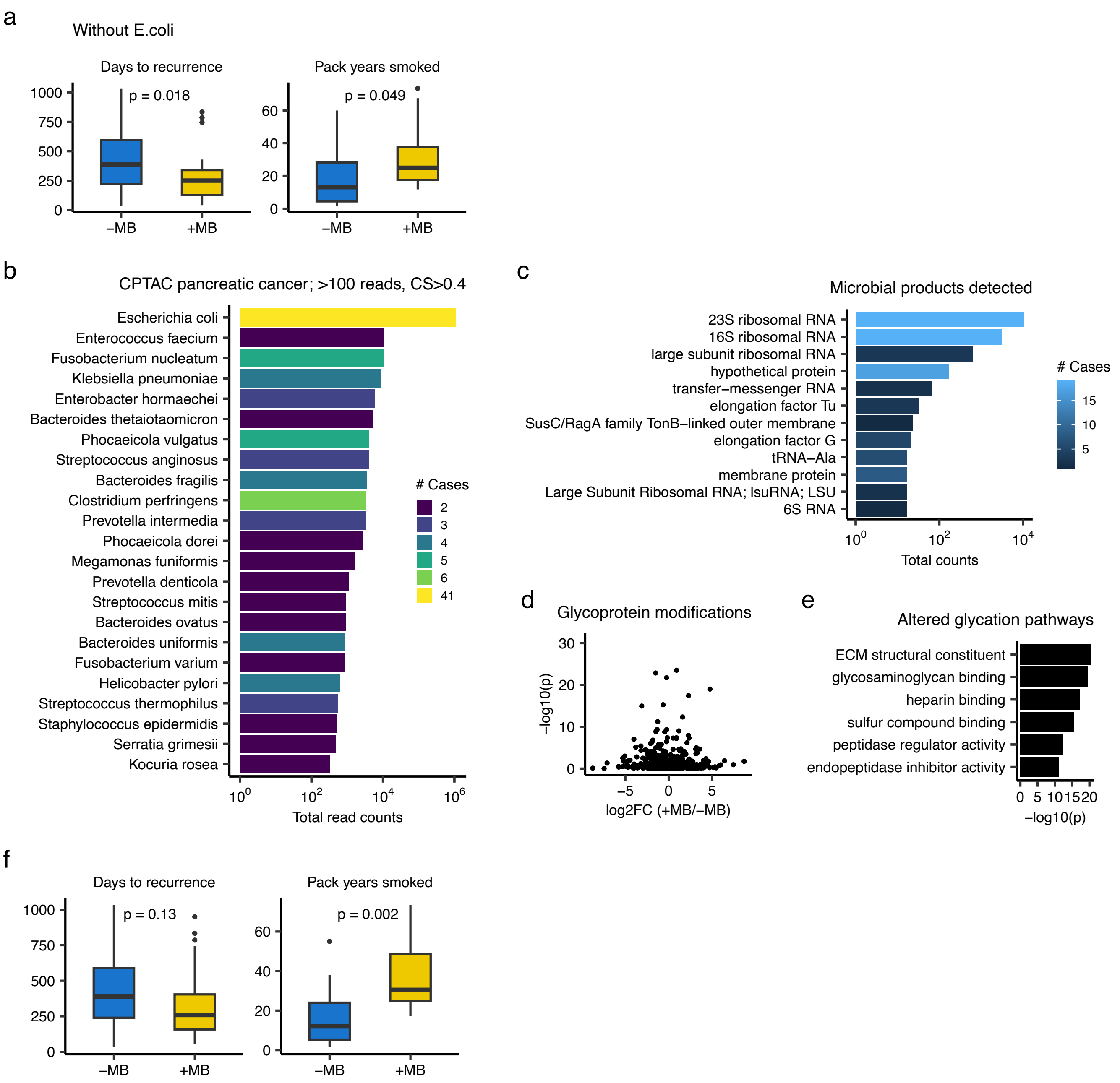

Supplement: 9 [file NIHPP2025.01.21.634087v1-supplement-9.pdf]
